# The relationships between burnout, general wellbeing, and psychological detachment with turnover intention in Chinese nurses: a cross-sectional study

**DOI:** 10.3389/fpubh.2023.1216810

**Published:** 2023-07-20

**Authors:** Fengzhi Zhang, Chunhui Lin, Xiaoxue Li, Manman Li, Ruolin Jia, Xiaoli Guo, Hua Bai

**Affiliations:** ^1^Department of Nursing, The Third Affiliated Hospital of Zhengzhou University, Zhengzhou, China; ^2^Department of Gynecology, The Third Affiliated Hospital of Zhengzhou University, Zhengzhou, China; ^3^Department of Reproduction, The Third Affiliated Hospital of Zhengzhou University, Zhengzhou, China; ^4^Department of Administration Office, The Third Affiliated Hospital of Zhengzhou University, Zhengzhou, China; ^5^Department of Infection Control, The Third Affiliated Hospital of Zhengzhou University, Zhengzhou, China

**Keywords:** psychological detachment, turnover intention, nursing, burnout, general wellbeing

## Abstract

**Background:**

It is critical to minimize nurse turnover to improve the quality of care and patient safety. In-depth investigation is required to better understand the factors related to nurses' turnover intentions.

**Aim:**

This study aimed to determine the relationships between burnout, general wellbeing, and psychological detachment with turnover intention among nurses in China.

**Methods:**

A cross-sectional survey using convenience sampling was conducted in one hospital in China between January 2023 and March 2023. A total of 536 nurses were surveyed using the General Wellbeing Schedule (GWB), the Maslach Burnout Inventory scale (MBI), the Psychological Detachment scale, and the Turnover Intention scale. The collected data were analyzed using SPSS 26.0 statistical software. The chi-square test and binary logistic regression analysis were used to explore the factors related to turnover intention.

**Results:**

Our data demonstrated that the turnover intention scores were 13 (10, 15.75), with 56% of nurses exhibiting a high level of turnover intention. Binary logistic regression analysis results indicated that being on a contract (OR = 4.385, 95% CI = 2.196–8.754), working in the pediatrics (OR = 2.392, 95% CI = 1.267–4.514) or obstetrics (OR = 2.423, 95% CI = 1.145–5.126) department, and experiencing burnout (OR = 1.024, 95% CI = 1.008–1.041) were associated with a heightened level of turnover intention. Conversely, organizational satisfaction (OR = 0.162, 95% CI = 0.033–0.787) and general wellbeing (OR = 0.967, 95% CI = 0.946–0.989) were identified as factors that hindered the intention to leave.

**Conclusions:**

Findings from this study suggest that nurses were employed on a contract basis, working in pediatric or obstetric departments, expressing dissatisfaction with the organization, reporting low general wellbeing, and experiencing high levels of burnout that require special attention. The identification of these risk factors can inform targeted interventions and support programs aimed at improving the wellbeing and retention of nurses in these settings.

## 1. Introduction

Nurses constitute the predominant occupational cohort in the healthcare sector, making up approximately 59% of the workforce; however, current trends show a projected shortage of 5.7 million nurses by 2030 (1), and nurse departures affect the global healthcare sector. In China, a rapidly aging population, nurse outflow, and an unbalanced distribution of the nursing workforce across the country make the shortage a long-term issue (2). A survey of the current WHO data reveals that China's nurse population density is still below the level of global distribution (3.75 nurses per 1,000 population) and much lower than that of developed countries, including the USA (14.6 nurses per 1,000 population) and the UK (8.2 nurses per 1,000 population) (1). Nursing staff shortages directly increase the workload of nurses in service, thereby negatively impacting job satisfaction and increasing the possibility of nurses exiting (3). In addition, patient safety and medical care quality are both affected by nurse staffing shortages, which also cut into the clinic's profit margin (4). While there are various factors attributed to the shortage of nurses, the principal factor is widely acknowledged to be the significant turnover of nursing staff (5). Nurses' turnover intention can significantly predict departure behavior (6). The likelihood that a nurse will leave his or her current organization or institution is defined as turnover intention (7). Nurses in different countries vary greatly in their degree of intention from 43% in Lebanon to 74% in Iceland (8). One study showed that a wide range of approximately 4% to 54% of nurses considered leaving the profession (9). The proportion of nurses in China who reported intention to leave ranged from 20% to 70% (10–12). Although much research has been conducted on the topic, the situation has clearly not improved, and more research is needed in this area to reduce nurse dropout rates (13).

Burnout significantly impacts nurse turnover (14), and high levels of burnout are a significant factor in nurses' turnover intention (15, 16). Burnout refers to chronic work stress that is not effectively managed, which in turn leads to emotional exhaustion, depersonalization, and low personal achievement, which is a common phenomenon in the healthcare profession (17). High burnout levels correlate with deterioration in safety and quality of care, patient satisfaction, and productivity (18, 19). Recognizing and controlling the factors associated with burnout is considered one of the strategies to reduce nurses' turnover intention (20, 21).

Nurse wellbeing is a vital factor in stabilizing the nursing workforce (22), and general wellbeing is a general psychological indicator of quality of life that reflects satisfaction with personal conditions (23). A favorable sense of wellbeing can improve nurses' psychological resilience and mental health and improve job satisfaction and performance (24, 25). According to findings, greater wellbeing is associated with lower absence and turnover intention and higher quality of care delivery (26). However, the impact on turnover intention from the perspective of general wellbeing has rarely been explored.

Moving away from work-related actions and thoughts is termed as psychological detachment from work, and it is regarded as an essential step in the healing process (27). Poor psychological detachment is strongly correlated with weariness, poorer sleep, and decreased wellbeing according to a prior study (28–30). Improving the level of psychological detachment from work can have a positive impact on patient safety, improve nurses' physical and emotional health, and enhance staff wellbeing (31, 32). Research indicates that psychological detachment can decrease nurses' turnover intention (33, 34), while research examining the impact of psychological detachment on turnover intention remains limited. This relationship requires further verification and in-depth exploration.

The characteristics of each job can be divided into work demands and resources based on the theory underlying the job demands–resources model (JD-R model) (35). In the workplace, job demands are the “negative variables” that drain an employee's energy, whereas job resources are the “positive factors” that make it easier to accomplish work objectives, lessen fatigue, and improve performance. Work resources become a motivational and supplementary way to mitigate the exhausting results of job demands and therefore significantly enhance employee wellbeing, which results in positive performance (36, 37). Based on the active process of the JD-R model, psychological detachment as a positive resource for individuals in the workplace helps restore individuals to their previous state during the process of burnout (38, 39), thus reducing nurses' turnover intentions. Other studies have demonstrated that psychological detachment from work might lessen stress related to work, which helps to prevent burnout (40, 41) and enhance nurses' wellbeing (30). Therefore, detachment from work and prevention of resource loss may increase nurses' willingness to stay in their jobs. However, few studies have examined the effects on nurses' turnover intentions from the perspective of psychological detachment and general wellbeing. Hence, this study aimed to explore the effects of burnout, general wellbeing, and psychological detachment on Chinese nurses' turnover intention.

## 2. Materials and methods

### 2.1. Design, setting, and participants

A cross-sectional survey using convenience sampling was conducted from January 2023 to March 2023 in a hospital in China. After being informed of the study's objectives, participants signed an informed consent form. The research was carried out using a paper questionnaire that included sociodemographic information, the General Wellbeing Schedule (GWB), the Maslach Burnout Inventory (MBI) scale, the Psychological Detachment scale, and the Turnover Intention scale. Finally, 536 eligible samples were analyzed for data. The inclusion criteria were being over age 18, having a nursing certificate from the People's Republic of China, working for more than a year, voluntarily engaging in the study, and providing complete informed consent. Nursing managers with the title of the head nurse or more, nurses working in administration, nurses on leave (sick leave, maternity leave, or marriage leave), and nurses suffering from serious illness were excluded.

### 2.2. Measurements

#### 2.2.1. Sociodemographic information of nurses

Based on the preliminary study and review (10, 42–44), a sociodemographic questionnaire was designed to collect participant characteristics, including sex, whether being the only child or not, age, marital status, children, educational background, professional title, level of nursing, position, the form of employment, years of service, night shift work, weekly working hours, monthly net income (CNY), and satisfaction with the institution.

#### 2.2.2. Turnover intention scale

We used the Chinese version of the 6-item Turnover Intention Scale, which was designed by Michael and Spector (45, 46), to evaluate participants' turnover intention as the outcome variable. The Turnover Intention Scale, which includes six items, covers three dimensions: one's probability of leaving a current job (2 items), the desire to look for another job (2 items), and the likelihood of finding a job beyond one's current sector (2 items). From “never” to “often,” all of the items were scored on a 4-point scale with values ranging from 1 to 4. The total points ranged between 6 and 24, and the turnover intention was stronger with a higher score. An entire average score of ≤1 indicated a very low desire for departure, >1 and ≤2 suggested a low desire to leave, >2 and ≤3 indicated a high desire to depart, and >3 indicated a very high desire to leave. The Chinese version of the Turnover Intention Scale showed good content validity (0.677) and reliability (Cronbach's alpha = 0.773) in its assessment (45). It is commonly used to measure nurses' intention to leave (42, 47). The Cronbach's alpha of the TIS-6 for the present study was 0.830.

#### 2.2.3. Maslach burnout inventory scale (MBI)

The 22-item Maslach Burnout Inventory Human Services Survey (MBI-HSS) was employed to assess job burnout levels in the participants (48). This scale contains three dimensions, including emotional fatigue (EE), depersonalization (DP), and decreased personal achievement, which are rated on a seven-point Likert scale, from 0 = never to 6 = every day. The cutoff points for high risk were >26 for emotional exhaustion, >9 for depersonalization, and <33 for reduced personal achievement. As an international standard scale, when a respondent's score on any dimension exceeds the critical value, burnout can be diagnosed (49). The Chinese version of the MBI-HSS Scale has been shown to have excellent credibility and validity (50, 51). Cronbach's alpha (0.916 in this study) was tested to assess the reliability of this scale.

#### 2.2.4. Psychological detachment from work

Sonnentag and Fritz constructed a four-item scale for assessing psychological detachment from work (38). The answers ranged from 1 (strongly disagree) to 5 (strongly agree) on a five-point Likert scale, with a sum score of 4 to 20 on the scale. Higher mean scores on the items suggest a greater level of psychological detachment. The Chinese version of the Psychological Detachment Scale, translated by Lu (34), demonstrated high reliability (α = 0.833) and satisfactory validity (*r* = 0.74) and has been employed in diverse studies (40, 52). The Cronbach's alpha score for this study was 0.909.

#### 2.2.5. General wellbeing schedule (GWB)

Developed by Fazio (53), translated and revised by a Chinese scholar (54), the General Wellbeing Schedule (GWB), which has 18 items and 6 dimensions, was used to evaluate nurses' subjective wellbeing and how satisfied they were with their overall lives. Six dimensions focus on measuring health concerns (10, 15), energy level (1, 14, 17), emotional–behavioral control (3, 7, 13), satisfying interesting life (6, 11), depressed/cheerful mood (2, 4, 12, 18), and anxiety level (5, 8, 9, 16). Items 1, 3, 6, 7, 9, 11, 13, 15, and 16 were reverse-scored. With a full score of 120, the higher the score was the greater the happiness. The scores corresponding to low, moderate, high, and excellent general wellbeing were 0–24, 25–48, 49–72, 73–96, and 97–120, respectively. Previous studies (25, 55) have confirmed the GWB's reliability and validity. Cronbach's alpha coefficient in this study was 0.877.

### 2.3. Data collection

**Figure 1:**
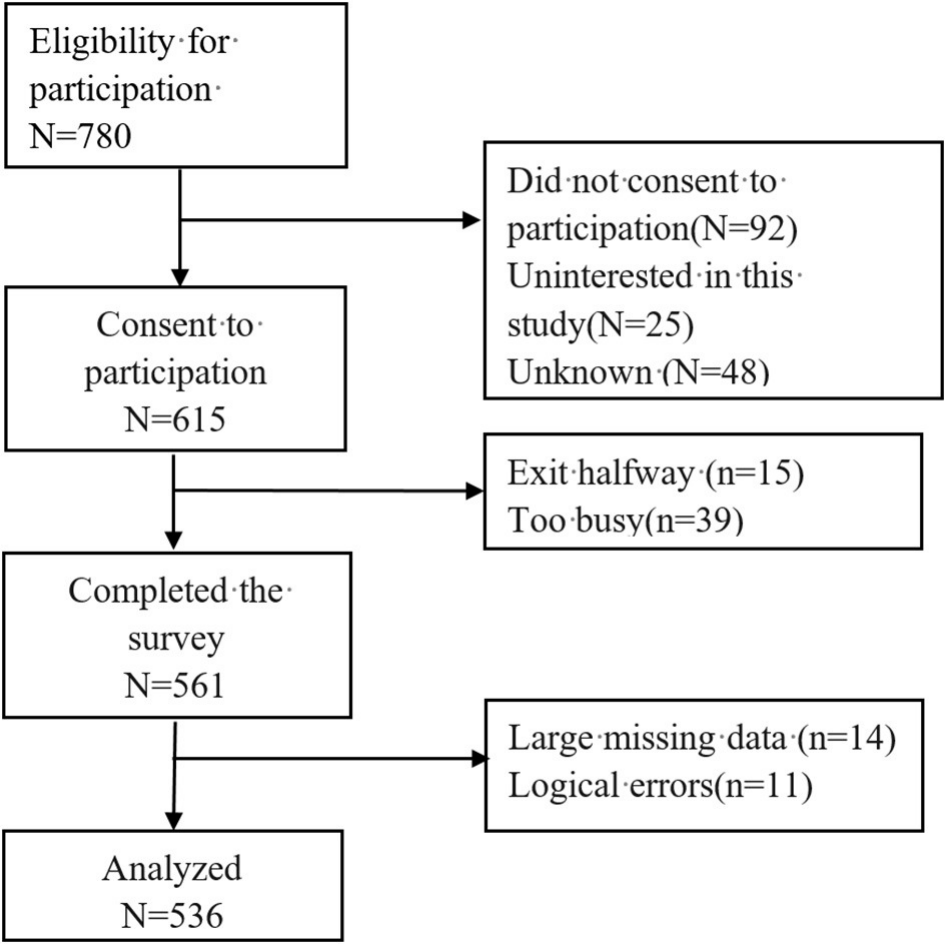
A flow chart of the participants.

This survey was conducted from January 2023 to March 2023 after being reviewed by the hospital ethics committee, and consent was obtained from the hospital nursing department and chief nursing officer. A paper questionnaire was distributed to the clinical nursing units of the hospital by trained research staff, who conducted face-to-face, one-on-one surveys with participants. Participants in the study signed a written consent form willingly and also acknowledged their participation. All questionnaires were completed independently and took approximately 12 min. Participants were guaranteed anonymity and confidentiality. Figure 1 depicts the participants' flowchart.

### 2.4. Data analysis

Frequency data were described using counts and percentages. The normality of continuous data was assessed using the Kolmogorov–Smirnov (K-S) test. Due to the non-normal distribution of variables, such as occupational burnout, general wellbeing, turnover intentions, psychological detachment total score, and scores for each dimension, descriptive statistics including the median and quartiles (M [Q25, Q75]) were utilized. Group comparisons were conducted using the chi-square test. Turnover intentions were treated as the dependent variable, with participants categorized into either the “low turnover intentions group” (Dimensional mean score ≤ 2) or the “high turnover intentions group” (Dimensional mean score >2). Pearson's chi-square test was employed to compare the two groups. Binary logistic regression analysis was performed to identify factors influencing turnover intention, with results presented as odds ratios (ORs) and 95% confidence intervals (CIs). Statistical significance was set at a *p* < 0.05 (two-tailed).

## 3. Results

### 3.1. Participant characteristics

A total of 536 valid questionnaires were analyzed. The participants were mainly women (95.1%) and married (70.3%). Most of them had obtained a bachelor's degree (90.1%), were at the intermediate or Junior (99.4%), and were general nurses (83.8%). Detailed information is shown in Table 1. In addition, the chi-square test results revealed statistically significant differences (*P* < 0.05) between the low turnover intention group and the high turnover intention group regarding variables such as age, marital status, children, professional title, nursing level, the form of employment, years of service, monthly night shifts, monthly net income (in RMB), ward of work, and satisfaction with the institution. Table 2 shows detailed information.

**Table 1:** Demographic characteristics of the participants (*n* = 536).

**Variable**	**Categories**	**Frequency (*n*)**	**Percent (%)**
Sex	Male	26	4.85%
	Female	510	95.15%
only child or not	Yes	77	14.37%
	No	459	85.63%
Age (years)	< 25	21	3.92%
	25–34	346	64.55%
	35–39	129	24.07%
	≥40	40	7.46%
Marital status	Unmarried	146	27.24%
	Married	377	70.34%
	Divorce or other	13	2.43%
Children	No	204	38.06%
	One	174	32.46%
	Two or more	158	29.48%
Educational background	Associate degree or below	34	6.34%
	Undergraduate	483	90.11%
	Graduate	19	3.54%
Professional title	Junior	237	44.22%
	Intermediate	296	55.22%
	Senior	3	0.56%
Nurse levels	N0	51	9.51%
	N1	88	16.42%
	N2	139	25.93%
	N3	187	34.89%
	N4	71	13.25%
Position title	General nurse	449	83.77%
	Responsible group leader	51	9.51%
	Chief Instructor	36	6.72%
Form of employment	Tenured nurse	221	41.23%
	Contract	125	23.32%
	Other	190	35.45%
Years of service (years)	< 5	96	17.91%
	5–9	175	32.65%
	10–20	233	43.47%
	>20	32	5.97%
Shifts per month in the past 6 months (days)	No	142	26.49%
	≤ 4	64	11.94%
	5–9	273	50.93%
	≥10	57	10.63%
Work hours per week in the past 1 month	≤ 35 h	15	2.80%
	36–40 h	261	48.69%
	**>40 h**	**260**	**48.51%**
Monthly net income (in CNY)	< 5,000	25	4.66%
	5,000–8,000	114	21.27%
	8,001–10,000	188	35.07%
	>10,000	209	38.99%
Ward of work	Gynecology	69	12.87%
	Obstetrics	71	13.25%
	Pediatrics	195	36.38%
	Special department (intensive care unit, surgery, emergency)	78	14.55%
	Others	123	22.95%
Satisfied with the current institution	Satisfied	291	54.29%
	Neutral	211	39.37%
	Dissatisfied	34	6.34%

**Table 2:** Univariate analysis of the participants (*n* = 536).

**Variable**	**Categories**	**Low (*N* = 236) *N* (%)**	**High (*N* = 300) *N* (%)**	***χ^2^***	***P* value**
Sex	Male	12 (46.2%)	14(53.8%)	0.050	0.823
	Female	224 (43.9%)	286 (56.1%)		
only child or not	Yes	37 (48.1%)	40 (51.9%)	0.590	0.442
	No	199 (43.4%)	260 (56.6%)		
Age (years)	< 25	1 (4.8%)	20 (95.2%)	16.163	0.001
	25–34	154 (44.5%)	192 (55.5%)		
	35–39	58 (45%)	71 (55%)		
	≥40	23 (57.5%)	17 (42.5%)		
Marital status	Unmarried	44 (30.1%)	102 (69.9%)	15.767	< 0.001
	Married	186 (49.3%)	191 (50.7%)		
	Divorce or other	6 (44%)	7 (56%)		
Children	No	71 (34.8%)	133 (65.2%)	13.431	0.001
	One	80 (46%)	94 (54%)		
	Two or more	85 (53.8%)	73 (46.2%)		
Educational background	Associate degree or below	11 (32.4%)	23 (67.6%)	3.378	0.185
	Undergraduate	214 (44.3%)	269 (55.7%)		
	Graduate	11 (57.9%)	8 (42.1%)		
Professional title	Junior	87 (36.7%)	150 (63.3%)	12.335	0.002
	Intermediate	146 (49.3%)	150 (50.7%)		
	Senior	3 (100%)	0 (0%)		
Nurse levels	N0	14 (27.5%)	37 (72.5%)	20.132	< 0.001
	N1	28 (31.8%)	60 (68.2%)		
	N2	58 (41.7%)	81 (58.3%)		
	N3	102 (54.5%)	85 (45.5%)		
	N4	34 (47.9%)	37 (52.1%)		
Position title	General nurse	199 (44.3%)	250 (55.7%)	1.486	0.476
	Responsible group leader	19 (37.3%)	32 (62.7%)		
	Chief Instructor	18 (50%)	18 (50%)		
Form of employment	Tenured nurse	118 (53.4%)	103 (46.6%)	43.931	< 0.001
	Contract	23 (18.4%)	102 (81.6%)		
	other	95 (50%)	95 (50%)		
Years of service (years)	< 5	27 (28.1%)	69 (71.9%)	21.067	< 0.001
	5–9	69 (39.4%)	106 (60.6%)		
	10–20	120 (51.5%)	113 (48.5%)		
	>20	20 (62.5%)	12 (37.5%)		
Shifts per month in the past 6 months (days)	No	80 (56.3%)	62 (43.7%)	13.273	0.004
	≤ 4	27 (42.2%)	37 (57.8%)		
	5–9	103 (37.7%)	170 (62.3%)		
	≥10	26 (45.6%)	31 (54.4%)		
Work hours per week in the past 1 month	≤ 35 h	3 (20%)	12 (80%)	4.048	0.132
	36–40 h	113 (43.3%)	148 (56.7%)		
	>40 h	120 (46.2%)	140 (53.8%)		
Monthly net income (in CNY)	< 5,000	3 (12%)	22 (88%)	10.920	0.012
	5,000–8,000	52 (45.6%)	62 (54.4%)		
	8,001–10,000	86 (45.7%)	102 (54.3%)		
	>10,000	95 (45.5%)	114 (54.5%)		
Ward of work	Gynecology	37 (53.6%)	32 (46.4%)	17.844	0.001
	Obstetrics	30 (42.3%)	41 (57.7%)		
	Pediatrics	69 (35.4%)	126 (64.6%)		
	Special department (intensive care unit, surgery, emergency)	30 (38.5%)	48 (61.5%)		
	others	70 (56.9%)	53 (43.1%)		
Satisfied with the current institution	Satisfied	177 (60.8%)	114 (39.2%)	78.175	< 0.001
	Neutral	57 (27%)	154 (73%)		
	Dissatisfied	2 (5.9%)	32 (94.1%)		

### 3.2. Burnout, subjective wellbeing, psychological detachment, and intention to leave scores of the study participants and their correlation analysis

The overall mean scores of burnout, psychological detachment, subjective wellbeing, and intention to leave were 43(28,58), 12(8,14), 73(65,81.75), and 13(10,15.75), respectively. Spearman's correlation analysis showed that the turnover intention was positively correlated with burnout (*r* = 0.479, *p* < 0.01) and significantly negatively correlated with general wellbeing (*r* = −0.399, *p* < 0.01) and psychological detachment (*r* = −0.091, *p* < 0.05). Burnout was significantly negatively correlated with psychological detachment (*r* = −0.152, *p* < 0.01) and general wellbeing (*r* = −0.657, *p* < 0.01), and psychological detachment was significantly positively correlated with general wellbeing (*r* = −0.284, *p* < 0.01), as detailed in Table 3.

**Table 3:** Spearman correlation analysis and descriptive statistics of main variables.

	**Score, *M* (*P*25, *P*75)**	**1**	**2**	**3**	**4**	**5**	**6**	**7**	**8**	**9**	**10**	**11**	**12**	**13**	**14**	**15**	**16**
1. Burnout (total score)	43 (28,58)																
2. Emotional fatigue	20 (14,27)	0.848^**^															
3. Depersonalization	5 (2,10)	0.813^**^	0.647^**^														
4. Decreased personal accomplishment	16 (9,23)	0.780^**^	0.422^**^	0.508^**^													
5. Psychological detachment (total score)	12 (8,14)	−0.152^**^	−0.234^**^	−0.047	−0.056												
6. General Wellbeing Schedule (total score)	73 (65, 81.75)	−0.657^**^	−0.692^**^	−0.447^**^	−0.445^**^	0.284^**^											
7. Energy level	14 (12,16)	−0.556^**^	−0.619^**^	−0.358^**^	−0.373^**^	0.271^**^	0.854^**^									
8. Health worry	7 (5,8)	−0.109^*^	−0.143^**^	−0.054	−0.04	0.055	0.216^**^	0.002									
9. Satisfying interesting life	6 (5,8)	−0.483^**^	−0.470^**^	−0.331^**^	−0.379^**^	0.110^*^	0.687^**^	0.590^**^	−0.022								
10. Depressed-cheerful mood	19 (16,22)	−0.586^**^	−0.596^**^	−0.412^**^	−0.420^**^	0.296^**^	0.887^**^	0.784^**^	0.031	0.570^**^							
11. Emotional-behavioral control	12.5 (11,14)	−0.502^**^	−0.457^**^	−0.370^**^	−0.378^**^	0.107^*^	0.671^**^	0.464^**^	0.039	0.484^**^	0.550^**^						
12. Relaxed vs tense-anxious	15 (13,17)	−0.522^**^	−0.585^**^	−0.346^**^	−0.323^**^	0.280^**^	0.819^**^	0.624^**^	0.216^**^	0.478^**^	0.648^**^	0.445^**^					
13.Turnover intention(total score)	13 (10, 15.75)	0.479^**^	0.476^**^	0.447^**^	0.296^**^	−0.091^*^	−0.386^**^	−0.332^**^	−0.075	−0.340^**^	−0.328^**^	−0.276^**^	−0.270^**^				
14. Possibility to resign from present job	4 (3,5)	0.512^**^	0.502^**^	0.442^**^	0.341^**^	−0.123^**^	−0.426^**^	−0.383^**^	–.088^*^	−0.368^**^	−0.382^**^	−0.285^**^	−0.305^**^	0.907^**^			
15. Motivation to seek another job	4 (2,6)	0.449^**^	0.431^**^	0.427^**^	0.297^**^	−0.076	−0.360^**^	−0.307^**^	−0.066	−0.328^**^	−0.303^**^	−0.259^**^	−0.261^**^	0.893^**^	0.766^**^		
16. Possibility to gained an external job	4 (4,5)	0.227^**^	0.247^**^	0.241^**^	0.098^*^	0.007	−0.156^**^	−0.112^**^	−0.001	−0.142^**^	−0.107^*^	−0.139^**^	−0.095^*^	0.687^**^	0.470^**^	0.410^**^	1

* p < 0.05, 2-tailed.

** p < 0.01, 2-tailed.

### 3.3. Factors associated with turnover intention

In the binary logistic regression analysis, variables identified as statistically significant (*p* < 0.05) through univariate analysis and Spearman's rank correlation analysis were included as independent variables. The binary logistic regression analysis indicated significant associations between contract employment (OR = 4.385, 95% CI = 2.196–8.754), pediatrics (OR = 2.392, 95% CI = 1.267–4.514) or obstetrics (OR = 2.423, 95% CI = 1.145–5.126), work satisfaction with the organization (OR = 0.162, 95% CI = 0.033–0.787), burnout (OR = 1.024, 95% CI = 1.008–1.041), and general wellbeing (OR = 0.967, 95% CI = 0.946–0.989), and the level of nurse turnover intentions (Table 4). Specifically, nurses with contract employment, working in pediatrics or obstetrics, dissatisfaction with the organization, high levels of occupational burnout, and low levels of general wellbeing exhibited higher levels of turnover intentions.

**Table 4:** A binary logistic regression analysis of factors associated with nurses' turnover intention (*n* = 536).

**Variables**	***B***	***OR***	***OR* (95% *CI*)**	***χ^2^* value**	***p*-value**
**Form of employment**					
Tenured nurse	Ref				
Contract	1.478	4.385	2.196–8.754	17.558	< 0.001
**Ward of work**					
Obstetrics	0.885	2.423	1.145–5.126	5.359	0.021
Pediatrics	0.872	2.392	1.267–4.514	7.242	0.007
others	Ref				
**Satisfied with the current institution**					
Satisfied	−1.822	0.162	0.033–0.787	5.095	0.024
Dissatisfied	Ref				
Burnout	0.024	1.024	1.008–1.041	8.742	0.003
General well-being	−0.033	0.967	0.946–0.989	8.555	0.003
Psychological detachment	−0.031	0.969	0.916–1.026	1.170	0.279
Hosmer-Lemeshow test	*P* = 0.395				

## 4. Discussion

In this study, the mean total score for nurses' turnover intentions was 13 (10, 15.75), 56% of nurses have a high level of turnover intention, and these results are consistent with international studies on the same issue (36.5% to 64.9%) (56–59) but are lower than similar findings in China (11, 42). The difference may be due to the timing of the survey in this study; nurses who had the intention to leave may have already left, thus yielding a sample with a lower intention to leave (60). In addition, our study also found that contract nurses had higher turnover intentions than tenured nurses, which is consistent with previous studies (61). Contract nurses are paid less and receive fewer benefits than tenured nurses even though they perform the same job duties. This suggests that nurses working under contract arrangements may experience unique challenges or job-related factors that increase their likelihood of considering alternative employment options. Further investigation into the specific aspects of contract employment, such as limited job security or reduced benefits, could provide a deeper understanding of its impact on turnover intentions (62). In line with previous studies' findings (45, 63), nurses in pediatrics and obstetrics are more inclined to leave their jobs than those in other departments. This may be because pediatric or obstetrics work demands more time and effort due to the specificity of the population they serve and the high demand for nursing skills (64). Exploring the specific stressors and work-related challenges faced by nurses in these areas could shed light on interventions and support systems needed to improve retention rates. The study also found that nurses' turnover intentions were significantly influenced by their satisfaction with the institution, and those who were satisfied with their institution had less intention to leave, which aligns with the results of the previous investigation (65). Dissatisfaction with various aspects of the work environment, such as leadership, organizational culture, workload, or professional development opportunities, may contribute to nurses' decision to seek employment elsewhere (13, 66). It is essential for healthcare organizations to proactively identify and address these areas of dissatisfaction to enhance nurse retention and overall job satisfaction.

The mean score for nurse burnout was 43 (28, 56) with average scores of 20 (14, 27) for emotional exhaustion, 5 (2, 10) for depersonalization, and 16 (9, 23) for low personal accomplishment. These findings are in line with previous studies by Chen R (67) and Karimi L (68), indicating that nurses are currently experiencing moderate levels of burnout. Importantly, these results also suggest that the impact of COVID-19 on nurses' mental health may have been underestimated (69). Additionally, this study's findings revealed that nurses' turnover intentions were positively correlated with burnout; the higher the degree of burnout was, the more likely they were to leave, similar to the results of existing studies (70, 71). Therefore, managers should monitor nurses' physical and emotional symptoms and establish emotional and social support networks (72), such as the Three Good Things based on WeChat (73), to improve nurses' psychological wellbeing and thus stabilize the nursing workforce.

The total general wellbeing score in this study was 73 (65, 81.75), suggesting that the nurses in this study had high general wellbeing, a result that was higher than other reports (74, 75) and lower than Iranian findings (76). In addition, this research found a significant negative effect of general wellbeing on turnover intentions, and these results are similar to those of previous studies (77–79), indicating that enhancing nurses' wellbeing can reduce their turnover intentions and turnover rates. Wellbeing is a positive psychological feeling and cognition; the stronger the happiness of nurses is, the better their psychological adaptability, which helps them maintain career stability (25). However, a meta-analysis (80) showed that the wellbeing of Chinese healthcare staff was on the decline. Therefore, measures such as positive psychology interventions can be taken to improve the wellbeing of nurses and thus reduce the turnover rate (81).

The total score of psychological detachment in this study was 12 (8, 14), which is a moderate level of psychological detachment, a result similar to the findings of Majeed's (82) study and Allen's research (52) and lower than other results (83). In addition, binary logistic regression analysis in this research found no statistically significant effect of psychological detachment on nurses' turnover intention, which is inconsistent with previous studies (33, 34). The disparities observed between our study and earlier research may be attributed to variances in cultural contexts and the diversity of sample sources. Further research is needed in the future to explore the relationship between psychological detachment and turnover intention.

## 5. Conclusion

The findings of this study indicated that among the 536 Chinese nurses surveyed, the prevalence of turnover intention was 56%. Satisfaction with the organization, employment on a contract basis, working in pediatrics or obstetrics departments, general wellbeing, and burnout are identified as significant predictors of nurses' turnover intention. Understanding these factors is crucial for developing targeted interventions to promote the stability of the nursing workforce.

### 5.1. Limitations of this study

There are several limitations to this study. First, this study uses a convenient sampling procedure that is considered direct, practical, and appropriate to the study's objectives but may have introduced bias. Second, using self-report questionnaires may have biased the results. Then, because only nurses from a tertiary hospital in Henan Province, China, were included in this study, the results may not be generalizable. Therefore, the number and scope of the study population need to be expanded for a comparative study. Third, since this survey was cross-sectional research, the results were unable to clarify the association of dependency among the factors involved and turnover intention. Finally, we recommend further research on the mediating models of psychological detachment, general wellbeing, burnout, and turnover intentions in the future, which will likely facilitate the development of related theories.

### 5.2. Implications for nursing management

The results of this study indicate the need to improve the working conditions and benefits of contract nurses to reduce the gap between contract and regular nurses. Creating a positive, supportive, and collaborative work environment is essential to increase nurses' organizational satisfaction. Providing professional training and support is essential to help pediatric or obstetric nurses meet the specific challenges and needs of their respective fields while considering improving the benefits package in the relevant departments. In addition, attention to the physical and mental health of nurses and the provision of stress management training and psychological counseling are important considerations.

## Data availability statement

The raw data supporting the conclusions of this article will be made available by the authors, without undue reservation.

## Ethics statement

The studies involving human participants were reviewed and approved by the Ethics Committee of the Third Affiliated Hospital of Zhengzhou University and the Chinese Health Department. The patients/participants provided their written informed consent to participate in this study.

## Author contributions

FZ contributed to editing the manuscript. CL participated in the drafting of the manuscript and survey design. XL participated in data collection. ML and RJ helped with data analysis and draft revision. The manuscript was examined by FZ, XG, and HB for quality and modifications were made in English. All authors contributed to the manuscript and approved the final version.
